# Selective MicroRNA Packaging Reveals Distinct Core Signatures in Human Mesenchymal-Stromal-Cell-Derived Extracellular Vesicles

**DOI:** 10.3390/ijms26147010

**Published:** 2025-07-21

**Authors:** Rachel E. Crossland, Clara Sanjurjo-Rodríguez, Monica Reis, Anne M. Dickinson, Elena Jones, Xiao-Nong Wang

**Affiliations:** 1 Translational and Clinical Research Institute, Faculty of Medical Sciences, Newcastle University, Newcastle upon Tyne NE1 7RU, UKanne.m.dickinson@alcyomics.com (A.M.D.);; 2 Institute of Biomedical Research of A Coruña (INIBIC), Centre of Advanced Scientific Researches (CICA), Universidade da Coruña, 15006 A Coruña, Spain; 3 Leeds Institute of Rheumatic and Musculoskeletal Medicine, University of Leeds, Leeds LS2 9JT, UK; e.jones@leeds.ac.uk

**Keywords:** extracellular vesicles, mesenchymal stromal cell, microRNA

## Abstract

Mesenchymal stromal cells (MSCs) have demonstrated therapeutic efficacy across numerous clinical applications, with evidence suggesting their paracrine effects, particularly through extracellular vesicles (EVs), possibly driving functional outcomes. In this study we perform the comprehensive characterization of microRNA expression profiles in human MSC-derived EVs (MSC-EV) compared to their parental cells, cultured under clinically relevant xeno-free conditions. MSCs were isolated from the bone marrows of healthy donors and characterised according to the International Society for Cellular Therapy criteria, while MSC-EVs were isolated using differential ultracentrifugation and validated according to the International Society for Extracellular Vesicle guidelines. NanoString profiling identified 590 mature microRNAs expressed across both populations, with 42 being significantly differentially expressed between MSC-EVs and parental MSCs. Five microRNAs were distinctly highly expressed in MSCs and five in MSC-EVs, while fifteen of the top twenty most abundant microRNAs showed high expression in both populations. MicroRNA expression patterns were validated in an independent cohort. Functional pathway analysis of differentially expressed microRNAs showed enrichment of key biological processes including cell proliferation, differentiation, and immune regulation. This standardised profiling approach develops our understanding of MSC/MSC-EV microRNA cargo, using a transparent methodological approach that allows for the improved comparability of datasets for the development and advancement of MSC-EV therapeutics.

## 1. Introduction

Mesenchymal stromal cells (MSCs) are defined as a heterogeneous population of multipotent progenitor cells that can proliferate and differentiate into tissues of mesenchymal origin, both in vivo and in vitro. They represent progenitor cells at various developmental stages of the mesodermal lineage, and have the capacity for self-renewal [1]. MSCs can be found in diverse adult and neonatal-derived tissues, such as the peripheral blood, adipose tissue, lung, heart, amniotic fluid, placenta, umbilical cord, and Wharton’s Jelly [2,3,4,5,6,7]. In order to be defined as an MSC, the International Society for Cellular Therapy (ISCT) proposed minimum criteria for the identification of human MSCs: (1) plastic adherence when cultured under standard conditions; (2) positive expression of CD105, CD73, and CD90 surface markers (>95% by flow cytometry), and negative expression of CD45, CD34, CD14 or CD11b, CD79a, CD19, or HLA-DR (human leukocyte antigen-DR) (≤2% by flow cytometry); and (3) the ability to differentiate into osteoblasts, chondrocytes, and adipocytes [8].

MSCs are easy to resuscitate and proliferate in vitro, enabling them to be mass-produced for their clinical application as regenerative agents to treat a variety of conditions [9]. In recent years, they have been the most studied stem cell type for clinical applications, and have played an effective therapeutic role in graft-versus-host disease (GvHD) [10], heart failure [11], liver failure [12], kidney injury [13], immune tolerance [14], osteoarthritis [15], diabetes [16], stroke [17], and Crohn’s disease [18], amongst others. With over 500 clinical trials of MSCs expected to be conducted by 2023 [19], the field has continued to expand rapidly. Recent clinical advances have demonstrated MSC efficacy in treating COVID-19-associated acute respiratory distress syndrome (ARDS), where allogenic MSC infusion in COVID-19 patients with ARDS proved to be safe and effective in decreasing inflammatory cytokines associated with a cytokine storm while efficiently improving clinical outcomes [20]. Meanwhile, umbilical-cord-derived MSCs have shown improved oxygenation profiles with no adverse effects as an adjunctive treatment for severe COVID-19 infection [21]. Demonstration that they can be transplanted without toxicity along with their clinical feasibility, practicality, and therapeutic efficacy in multiple animal models has given rise to a series of broad clinical trials. The grounds for many clinical applications of MSCs can be attributed to their immunomodulatory effects on both the innate and adaptive immune systems. Indeed, they can skew mature dendritic cells (DCs) to an immature state, diminish natural killer (NK)-cell cytotoxicity, dampen neutrophil respiratory burst activity, impair B-cell differentiation, and inhibit T-cell proliferation in vitro [22,23,24,25,26].

However, despite the beneficial properties of MSCs as a cellular therapy, a lack of correlation has been observed between functional improvement and engraftment, as well as differentiation at the tissue injury site. This has led to the ‘paracrine hypothesis’, where it is thought that it is not the MSCs themselves, but rather their secretome that is driving therapeutic efficacy, via paracrine activity. Indeed, MSCs produce a plethora of biologically active molecules and soluble factors that mediate their anti-proliferative and anti-inflammatory properties, including microvesicles [27], microparticles [28], and exosomes [29] that are enriched in bioactive molecules including lipids, proteins, mRNA, tRNA, lncRNA, microRNA, and mitochondrial DNA [30]. Collectively, these extracellular vesicles (EVs) have been proposed to interact with heterogeneous cell types, as a communication vesicle to maintain a dynamic and homeostatic tissue environment [27,31].

Extracellular vesicles offer a number of therapeutic advantages over their parental cells [32]. They are non-self-replicating, thus avoiding the risk of tumorigenicity [33]; they have both biocompatibility and low immunogenicity, enabling them to cross-protective barriers [34]; production can be upscaled via immortalised cells to achieve a higher potency [35]; they protect their internal cargo via their robust lipid membrane structure, allowing their ease of use [35]; and their encapsulation allows the potential for specific drug loading [36].

Recent advances in microRNA profiling have begun to elucidate the molecular cargo of MSC-EVs, revealing both conserved and variable microRNA signatures across different studies. Several investigations have identified core miRNA populations in MSC-EVs, with miR-21, miR-125b-5p, miR-199a-3p, and let-7 family members consistently being detected across multiple MSC sources [37,38,39,40]. Notably, comparative analyses have revealed selective microRNA packaging, where certain microRNAs are enriched in EVs relative to their parental cells (such as miR-451a, miR-630, and miR-638), while others remain predominantly cellular (including miR-125b and miR-34a-5p) [37,38]. These patterns suggest active sorting mechanisms rather than passive microRNA transfer, with functional implications for the therapeutic targeting of regeneration, apoptosis, and inflammation pathways [41]. However, significant inconsistencies persist across studies, with the proportion of shared microRNAs between MSCs and EVs ranging from 38.7% to 98.3% depending on the experimental conditions [40,42]. Furthermore, most existing studies have utilised foetal bovine serum (FBS)-containing media for MSC culture and EV production [38,41,43], potentially introducing xenogeneic contamination that could confound clinical translation.

To culture-expand isolated MSCs for therapeutic use, basal culture medium is frequently supplemented with FBS [44]. However, FBS contains high levels of endotoxins, is a potential source of microbial contaminants and bovine protein contaminants, and xenoantigen *N*-glycolylneutraminic acid (Neu5Gc) in FBS can be taken up by MSCs, potentially provoking immunological reactions and post-transplantation MSC lysis [45,46]. Critically, FBS-derived EVs and microRNAs may contaminate MSC-EV preparations, potentially masking true MSC-derived microRNA signatures and limiting the reproducibility of functional studies. Thus, the generation of MSCs for clinical applications should comply with Good Manufacturing Practice (GMP) and avoid the use of animal sera. Human platelet lysate (hPLT) has been proposed as a replacement for FBS, enhancing MSC proliferation kinetics ex vivo whilst maintaining the MSC phenotype, chromosomal stability, and immunoprivileged status [47,48,49]. However, comprehensive microRNA profiling of MSC-EVs derived under xeno-free conditions remains limited, representing a critical knowledge gap for clinical translation.

Despite the significant advances in the development of MSC products, relatively few have been approved for market to date, and discrepancies and controversies about the biology, functions, and potency of MSC-EVs have arisen. This may be attributed to several factors, including the heterogeneity of MSCs and their preparation, differing methods of MSC-EV production and separation, a lack of standardised quality assurance assays, and the limited reproducibility of in vitro and in vivo functional assays [50], as highlighted by a review of existing MSC-EV microRNA profiling studies (Table S1). While existing microRNA profiling studies have provided valuable insights into the molecular composition of MSC-EVs, several critical gaps remain: (1) the limited paired comparisons between MSCs and EVs under standardised conditions, (2) the predominant use of xenogeneic culture systems that may confound microRNA signatures, and (3) the lack of consensus on core therapeutic microRNA signatures across different MSC sources and culture conditions. To address these challenges, we conducted a comprehensive assessment of the microRNA repertoire from human bone-marrow-derived MSCs (BM-MSCs) obtained from healthy donors and cultured under clinically relevant, xeno-free conditions using hPLT. By simultaneously profiling parent MSCs and their derived EVs under rigorously defined conditions, this study reveals critical insights into therapeutic microRNA signatures while establishing standardised benchmarks that advance the understanding of molecular mechanisms and accelerate clinical translation.

## 2. Results

**Figure 1:**
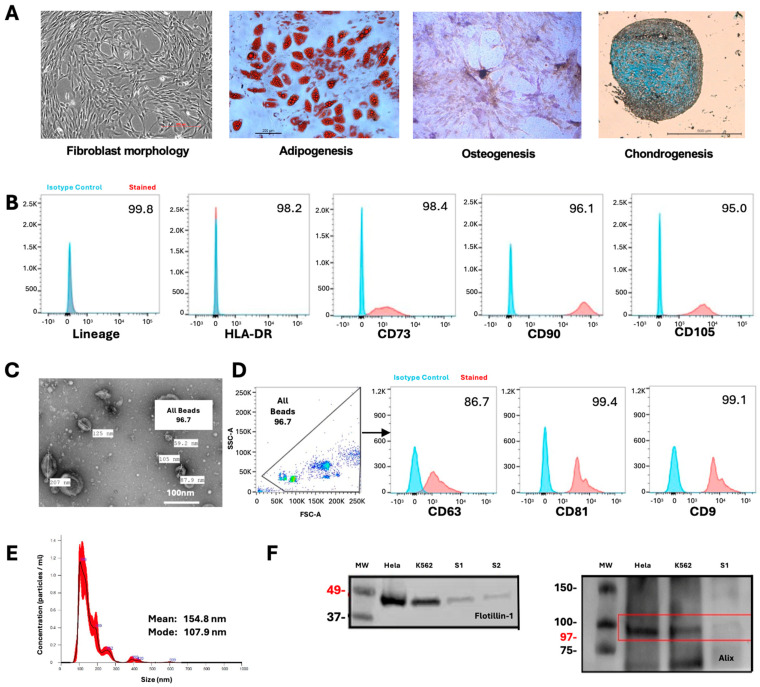
Characteristics of MSCs and MSC-EVs. (**A**) In accordance with ISCT guidelines, MSCs demonstrated expected morphology and tri-lineage differentiation potentials. Representative images from left to right: phase contrast image showed a typical fibroblast-like morphology; positive Oil Red O staining showed extensive lipid vacuole formation, a typical feature of adipogenesis; positive Alkaline Phosphatase/von Kossa staining indicated osteogenic differentiation; positive Alcian Blue staining indicated formation of proteoglycan, a characteristic feature of chondrogenesis. Scale bars indicate 200, 200, 500, and 500 μm, respectively. (**B**) In accordance with ISCT guidelines, the MSC surface phenotype was <2% positive for lineage-specific markers CD14, CD19, CD34, CD45, and HLA-DR, while being >95% positive for the expression of CD105, CD73, and CD90. (**C**) Transmission electron microscopy showing isolated MSC-EVs of the expected size range (30–200 nm) and morphology (cup-shaped vesicles). (**D**) Bead-based flow cytometry data showing positive expression of tetraspanins CD63, CD81, and CD9 on isolated extracellular vesicles. EVs were isolated and coated onto aldehyde/sulfate latex beads prior to staining. The FSC vs. SSC gating included all EV coated beads and excluded debris prior to analysing individual markers. Data are shown as overlay histograms; the percentage of positive beads is indicated in each plot. EVs demonstrated high surface expression of all three markers, confirming successful isolation and surface phenotyping. (**E**) Nanoparticle tracking analysis based on NanoSight, indicating serum MSC-EV size distribution within the expected range (mode 108.9 nm). (**F**) Western Blot analysis of positive EV markers of Flotillin-1 and Alix expression.

Bone-marrow-derived MSCs were cultured from healthy donor aspirates through standard plastic adherence protocols and validated according to the ISCT guidelines [8]. Characterization confirmed the MSC identity through multiple criteria: cells exhibited a typical spindle-shaped morphology and demonstrated a multipotent differentiation capacity across three lineages—adipogenic, osteogenic, and chondrogenic pathways (Figure 1A). Flow cytometric analysis verified the expected immunophenotype, with minimal expression (<2%) of hematopoietic and immune markers (CD14, CD19, CD34, CD45, HLA-DR) and positive expression (>95%) of mesenchymal markers CD105, CD73, and CD90 (Figure 1B).

For EV isolation, MSC-conditioned media were collected under serum-free conditions and processed via sequential differential ultracentrifugation. The resulting MSC-EVs underwent comprehensive characterization following the ISEV recommendations [51]. Transmission electron microscopy revealed the characteristic cup-shaped vesicular ultrastructure (Figure 1C). Flow cytometric evaluation confirmed the expression of EV markers CD63, CD81, and CD9 (Figure 1D). Nanoparticle tracking analysis demonstrated a size distribution consistent with the expected EV diameter range, with the modal particle size falling within established parameters (Figure 1E). Western blot analysis further validated EV identity through detection of the signature markers Flotillin-1 and Alix (Figure 1F).

### 2.1. MSC and MSC-EV Demonstrate High Expression of Selected MicroRNAs

**Figure 2:**
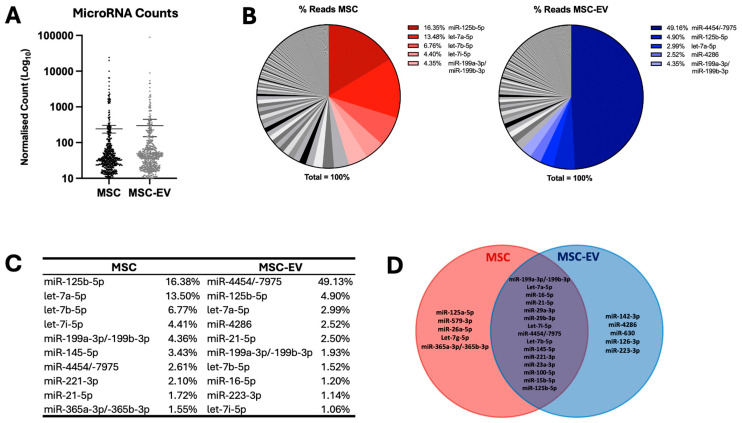
MicroRNAs highly expressed in MSCs and MSC-EVs. (**A**) Scatter graphs to show the number of microRNAs expressed in MSCs vs. MSC-EVs. MicroRNA data are expressed as normalised NanoString counts (Log_10_ scale). MSCs are depicted in black and MSC-EVs in grey. Error bars represent mean +/− standard error of the mean (SEM). (**B**) Pie charts show the percentage counts for microRNAs profiled in MSCs and MSC-EVs. The top five most highly expressed microRNAs are highlighted in colour, and percentage NanoString counts are shown (normalised counts). (**C**) Table detailing the top 20 most highly expressed microRNAs in MSCs and MSC-EVs. The percentage NanoString counts are shown for each microRNA. (**D**) Venn diagram demonstrating the top 10 most highly expressed microRNAs in MSCs (red) and MSC-EVs (blue), and the extent of overlap between these two groups.

We molecularly profiled the microRNA composition of n = 4 independent hPLT cultured MSC-EVs in comparison to their parental MSC cells (n = 4) using NanoString technology and observed expression of n = 590 mature microRNAs after filtering (microRNA expressed above LOD in ≥2 samples). Overall, there was no significant difference in the number of microRNAs expressed in the MSCs vs. MSC-EVs (MSC total = 147,272 vs. MSC-EV total 180,481; MSC Average = 250 vs. MSC-EV Average = 306) (*p* > 0.05) (Figure 2A). There was no significant difference in microRNA counts between individual MSCs or MSC-EV samples (*p* < 0.05).

Assessing the most abundant microRNAs in each population, the five most highly expressed microRNAs in MSCs comprised 45% of all reads (miR-125b-5p: 16.35%, let-7a-5p: 13.48%, let-7b-5p: 6.76%, let7i-5p: 4.40%, and miR-199a-3p/-199b-3p: 4.35%), while the five most highly expressed microRNAs in MSC-EVs comprised 63.9% of all reads (miR-4454/-7975: 49.16%, miR-125b-5p: 4.90%, let-7a-5p: 2.99%, miR-4286: 2.52%, miR-199a-3p/-199b-3p: 4.35%) (Figure 2B,C).

Focusing on the top 20 most highly expressed microRNAs in each population on average, there were five microRNAs that were distinctly highly expressed in MSCs and five in MSC-EVs, and 15 of the top 20 showed high expression in both MSCs and MSC-EVs (Figure 2D).

### 2.2. MSC-EVs Demonstrate Distinct MicroRNA Profiles from Parental MSCs

**Figure 3:**
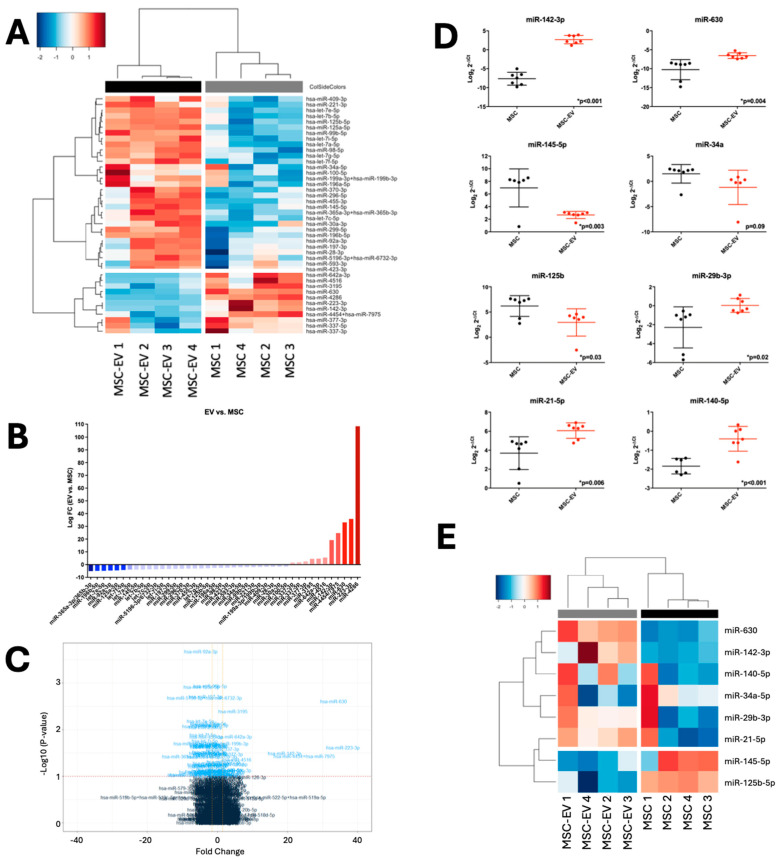
Differential microRNA expression and validation in MSCs vs. MSC-EVs. (**A**) Unsupervised hierarchical clustering of significantly differentially expressed microRNAs (*p* < 0.05, n = 42), based on normalised digital expression counts, in MSCs vs. MSC-EVs. Each column represents an individual sample. Relative expression changes are indicated by the colour key (red: high; blue: low). MSC-EVs are depicted by black shading, while MSCs are depicted by grey shading. (**B**) Bar plot to show fold change differences between significantly differentially expressed microRNAs (*p* < 0.05, n = 42). Relative expression changes are indicated by the colour key (red: high; blue: low). (**C**) Volcano plot to show microRNA expressed in MSCs vs. MSC-EVs. Significantly differentially expressed microRNAs between the two groups are depicted in lighter blue (*p* < 0.05). The red dashed line denotes *p*-value threshold *p* < 0.05, while the orange dashed lines denote fold change (FC) threshold of −1.5/1.5. (**D**) Validation of differential microRNA expression between MSCs vs. MSC-EVs by qRT-PCR in an independent cohort (n = 7 paired samples). Expression differences between groups were calculated using the students paired *t*-test. Error bars indicate mean +/− SEM. (**E**) Unsupervised hierarchical clustering of qRT-PCR validated microRNAs according to their NanoString expression data (n = 8 microRNA, n = 4 paired samples), based on normalised digital expression counts, in MSCs vs. MSC-EVs. Each column represents an individual sample. Relative expression changes are indicated by the colour key (red: high; blue: low). MSC-EVs are depicted by black shading, while MSCs are depicted by grey shading.

Of the n = 590 mature microRNAs expressed in MSCs and their derived MSC-EVs, 42 microRNAs were significantly differentially expressed (DE) between the two populations (fold change (FC) range = −5.09–108.37, *p*-value range *p* < 0.001–0.048). This comprised 31 downregulated microRNAs (Log_2_ FC range = −1.57–5.09, *p*-value range *p* < 0.001–0.038) and 11 that were upregulated (FC range = 1.5–108.37, *p*-value range *p* < 0.001–0.048) in MSC-EVs compared to MSCs (Figure 3A–C).

### 2.3. MSC and MSC-EV MicroRNA Profiles Can Be Validated in Independent Cohorts

To validate microRNA expression profiles, five significantly DE microRNAs between MSCs and their derived MSC-EVs were confirmed by qRT-PCR in an independent cohort of n = 7 paired samples. Significant differential expression was observed, thus validating the NanoString data (upregulated in MSC-EVs: miR-142-3p *p* < 0.001, miR-630 *p* = 0.004; downregulated in MSC-EVs: miR-145-5p *p* = 0.003, miR-34a *p* = 0.09, miR-125b *p* = 0.03) (Figure 3D). Similarly, 3 microRNAs identified as being within the top 20 most abundantly expressed in MSC-EVs and MSCs were further validated in the same cohort (miR-29b-3p *p* = 0.02, miR-21-5p *p* = 0.006, miR-140-5p *p* < 0.001) (Figure 3D). The selected validation microRNAs could effectively segregate MSC-EVs from parental MSCs according to non-supervised hierarchical clustering of NanoString count data (Figure 3E).

### 2.4. MSC-EV MicroRNAs Are Predicted to Target Fundamental Pathways

**Figure 4:**
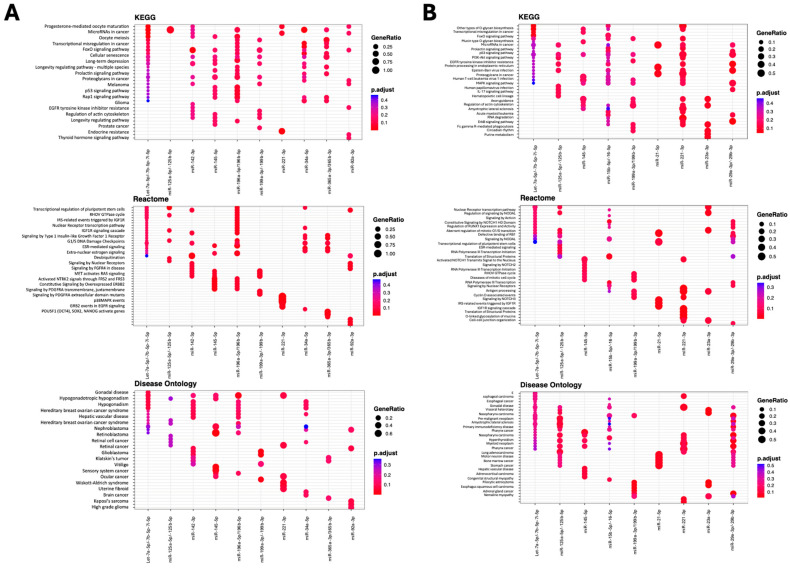
Differential MicroRNA MIENTURNET expression functional enrichment analysis. KEGG pathway, REACTOME pathway, Disease Ontology, and WikiPathway analysis of differentially expressed microRNA targets, generated according to the MIENTURNET package. (**A**) The top 20 most significant functional enrichment interactions for 42 microRNAs differentially expressed between MSCs and MSC-EVs. (**B**) The top 20 most significant functional enrichment interactions for 15 microRNAs that were similarly highly expressed in both MSCs and MSC-EVs. Dot plots are shown for each analysis, where the Y-axis reports the annotation categories and the X-axis reports the miRNA family with the number of recognised targets in round brackets (i.e., target with at least one annotation). The colours of the dots represent the adjusted *p*-values (FDR), whereas the size of the dots represents gene ratio (i.e., the number of miRNA targets found annotated in each category over the total number of recognised targets indicated in round brackets).

The 42 significantly DE microRNAs were investigated using MIENTURENET, to search for microRNA–target interactions. TargetScan target enrichment analysis, filtered to a minimum of four interactions, identified 262 target genes. Functional enrichment was performed based on the top 10 microRNA families according to the number of interactions (let-7a-5p/-7b-5p/-7c-5p/-7e-5p/7f-5p/-7g-5p/-7i-5p/miR-98-5p = 35; miR-34a-5p = 17; miR-125a-5p/-125b-5p = 14; miR-199a-3p/-199b-3p = 6; miR-142-3p = 9; miR-196a-5p/196b-5p = 10; miR-92a-3p = 8; miR-221-3p = 8; miR-145-5p = 8; miR-365a-3p/-364b-3p = 7) resulted in 51 microRNA target genes mapping to 138 KEGG pathways and 50 genes relating to 368 REACTOME entries, while 46 genes were associated with 379 Disease Ontologies. The top 20 most significant functional enrichment interactions are shown in Figure 4A.

Focusing on MSC-EV microRNAs that may drive the therapeutic efficacy of the parental MSCs, we also performed microRNA–target interaction analysis for the 15 microRNAs that were similarly highly expressed in both MSCs and MSC-EVs (within the top 20 that were the most highly expressed) (see Figure 2D). MIENTURENET (based on TargetScan filtered to a minimum of four interactions), identified 275 target genes. Functional enrichment was performed based on the top 10 microRNA families according to the number of interactions (let-7a-5p/-7b-5p/-7i-5p = 30; miR-125b-5p = 14; miR-145-5p = 10; miR-15b-5p/-16-5p = 30; miR-199a-3p/-199b-3p = 11; miR-21-5p = 4; miR-221-3p = 8; miR-23a-3p = 6; miR-29a-3p/-29b-3p = 16) resulted in 65 microRNA target genes mapping to 175 KEGG pathways and 392 REACTOME entries, while 80 genes were associated with 361 Disease Ontologies (Figure 4B).

## 3. Discussion

In this study, we have defined distinct microRNA expression profiles from bone-marrow-derived MSCs and their derived MSC-EVs, focusing on both shared and divergent expression profiles and their potential regulatory mechanisms. Our findings highlight key mechanisms by which MSC-EVs may exert therapeutic advantages over their parental cells, driven by their unique microRNA cargo. This study extends the current understanding of MSC-EV microRNA biology by addressing key methodological limitations in the field, and introducing more robust characterization and validation. Thus, our results contribute to more advanced mechanistic insight and translational relevance in the field of MSC-EV research.

Consistent with the recommended International Society for Extracellular Vesicle (ISEV) guidelines, we performed comprehensive MSC-EV characterization using flow cytometry, transmission electron microscopy, and nanoparticle tracking analysis. This confirmed the presence of typical EV features, including a cup-shaped morphology, expression of EV-specific markers (CD63/CD81/CD9, Flotillin-1, Alix), and a size distribution peaking within the expected EV range (100–200 nm) [51]. This rigorous characterization strengthens the validity of our downstream MSC-EV microRNA profiling. In contrast, many previous MSC-EV studies have relied on limited or incomplete EV characterization, raising the possibility of potential contamination with non-EV particles and the misidentification of vesicle populations, and thus impacting the reproducibility and comparability of functional and molecular findings.

Our microRNA profiling identified miR-125b-5p as being the most highly expressed microRNA in MSCs and second most highly expressed microRNA in MSC-EVs, aligning with several previous studies [37,52,53,54]. This consistency suggests that certain microRNAs, such as miR-125b-5p and members of the let-7 family, represent a core MSC signature. Indeed, we observed that the top five microRNAs in MSCs (miR-125b-5p, let-7a-5p, let-7b-5p, let-7i-5p, and miR-199a-3p/-199b-3p) constitute 45% of all reads, closely parallelling previous findings [38,39,53,54,55,56,57]. This consistency suggests these microRNAs may also contribute to a core MSC signature, regardless of culture conditions or the MSC source.

In contrast, MSC-EVs showed a striking enrichment of miR-4454/7975, constituting 49.16% of all reads. This observation, which aligns with that of Vaka et al. [52], supports the concept of selective microRNA packaging into EVs rather than passive cellular sampling, as also observed by Figueroa et al. [58] and Zubkova et al. [59]. Our finding that over 50% of EV microRNA reads are derived from the top five microRNAs supports the “functional miRNA subset” hypothesis [60], potentially explaining how low-copy microRNAs achieve therapeutic effects.

While 15 of the top 20 microRNAs were shared between MSCs and MSC-EVs, our comparative profiling identified the unique enrichment of miR-4454/-7975 (49% of reads) and the significant differential expression of 42 microRNAs (31 downregulated, 11 upregulated). These patterns are consistent with ESCRT-dependent or -independent sorting mechanisms [59] and parallel findings from Phinney et al. [37], who reported the differential expression of 156 microRNAs between MSCs and their EVs. Our data strengthens the evidence that MSC-EVs do not simply mirror the microRNA profile of their parental cells, but instead act as precision delivery vehicles that selectively package regulatory RNAs for targeted biological effects.

Our bone-marrow-derived MSC findings demonstrate remarkable conservation with alternative MSC sources, particularly adipose tissue (AT) and umbilical cord (UC) MSCs, which are increasingly favoured clinically due to their accessibility and expansion potential. Despite source-specific quantitative differences, such as our miR-4454/-7975 dominance (49.16%) versus miR-486-5p enrichment in AT-MSC-EVs, core therapeutic microRNA signatures are conserved across tissue origins. The UC-MSC-EV profiling of Vaka et al., under similar xeno-free conditions, identified miR-4454+miR-7975 and miR-125b-5p as being among the highest expressed microRNAs [52], directly paralleling our findings. Similarly, multiple studies across BM, AT, and UC sources consistently report the high expression of miR-21-5p, miR-125b-5p, miR-29b-3p, and let-7 family members [39,54,61], all of which were validated in our study. Zhou et al. identified 91 commonly detected microRNAs across four MSC tissue sources [54], with substantial overlap with our top 20 abundant microRNAs, while Soni et al. demonstrated that 122 microRNAs were expressed across BM, AT, and Wharton’s jelly MSC-EVs [40]. This cross-source conservation suggests that therapeutic mechanisms identified in our BM-MSC-EVs are likely translatable to the more clinically tractable AT and UC sources, supporting their continued clinical development. Furthermore, microRNAs identified by Sanjurjo-Rodríguez et al. as being highly expressed in MSC/MSC-EVs derived from the bone marrow of patients with osteoarthritis also showed considerable overlap with those in the present study, including the MSC-EV expression of miR-142-3p, miR-4286, miR-630, and miR-223-3p [62]. This suggests that MSC-EV cargo-loading mechanisms may remain consistent, despite an altered pathological microenvironment.

Functional analysis of differentially expressed microRNAs between MSCs and MSC-EVs revealed several candidates that likely contribute to the distinct biological actions of MSC-EVs. Upregulated microRNAs such as miR-142-3p and miR-630 are known to modulate immunological and stress-response pathways [63,64], while downregulated microRNAs like miR-145-5p and miR-125b-5p are involved in osteogenic differentiation, suggesting that their retention in MSCs helps reserve stemness.

Further pathway enrichment analysis revealed that the differentially expressed microRNAs regulate critical signalling networks, including PI3K-Akt, TGF-B, and FoxO pathways. For example, miR-21-5p and miR-29b-3p, validated in both experimental cohorts, modulate extracellular matrix remodelling, a mechanism that is critical to bone regeneration [65]. Similarly, the let-7 family and miR-34a-5p target overlap with the KEGG FoxO, PI3K-Akt, and TGF-β pathways, while miR-199a-3p and miR-221-3p are linked to fibrosis and metabolic regulation. These findings underscore the capacity of MSC-EVs to orchestrate complex cellular responses via a well-defined microRNA cargo.

In addition to highlighting differential microRNA expression, our study also focussed on microRNAs that are highly expressed in both MSC and MSC-EV populations. The shared abundance of pro-differentiation microRNAs such as miR-145-5p and miR-125b-5p in MSCs suggests active retention to maintain lineage potential, while immunomodulatory microRNAs such as miR-142-3p are preferentially exported. This supports a refined model of selective microRNA packaging, where specific microRNAs are retained or secreted based on their role in maintaining MSC function or enhancing EV-mediated effects [59]. Our pathway level insights further reinforce that MSC-EVs carry microRNAs targeting immunomodulatory and regenerative processes, such as miR-142-3p and let-7a-5p (TLR4 and TNF-α [66]), miR-21-5p and miR-29b-3p (collagen remodelling [67,68]), and miR-620 (apoptosis suppression [69]).

Importantly, our results reveal that co-enriched microRNAs (e.g., miR-21-5p and miR-29b-3p) converge on common targets such as extracellular matrix pathways, supporting a model of synergistic microRNA action. This combinatorial targeting may underline the broad therapeutic potential observed with MSC-EV treatments, enhancing the therapeutic benefits attributed to their protein or lipid content.

One of the key strengths of this study lies in its methodological rigor, which addresses key limitations identified across the literature (Table S1). Indeed, inconsistencies in previous studies, including variations in microRNA analysis techniques, EV isolation approaches, and characterization standards, in addition to a lack of validation, have limited comparability across the field. We addressed these challenges by employing robust methods and providing comprehensive EV characterization. The use of NanoString technology enabled robust profiling despite a low RNA input, consistent with advancements in EV miRNA detection [70], and overcomes some of the limitations of RNA-seq or microarrays used in prior studies [60,71]. Furthermore, we performed qRT-PCR in an independent cohort to validate NanoString data, addressing a critical limitation in EV research, where small sample sizes often hinder reproducibility. While many earlier studies used FBS-supplemented media for MSC cultures (Table S1) [38,43,72], our study was conducted using hPLT, aligning with Good Manufacturing Practice for clinical applications. By employing xeno-free conditions (used in very few prior studies (Table S1) [52,73], comprehensive ISEV-compliant characterization, and the paired analysis of MSCs and EVs, our study provides more clinically relevant and reproducible findings.

While our findings do not introduce an entirely novel mechanistic paradigm, this study makes an important contribution by addressing key translational and methodological challenges in the field of MSC-EV research. Notably, we employed xeno-free, GMP-compatible conditions using hPLT and adhered to ISEV-compliant EV characterization standards, which remain inconsistently applied across the literature (Table S1). A small number of previous studies have incorporated xeno-free MSC culture conditions [52,73]; however, there remains inconsistency in the use of human MSCs and rigorous EV characterization between these studies. Furthermore, we included a cross-cohort validation of key microRNAs via qRT-PCR, which was absent in many prior studies. By generating reproducible, high-confidence microRNA profiles under clinically relevant conditions, our work establishes a standardised reference framework that can support future therapeutic applications and mechanistic studies. In a field where variability in culture conditions, EV isolation, and miRNA profiling techniques often limits comparability, our study offers ab incremental advancement in both scientific rigor and translational readiness.

In conclusion, our study demonstrates that MSC-EVs carry a selectively enriched and functionally potent microRNA cargo, distinct from their parental MSCs. These microRNAs are enriched for regulatory pathways central to immunomodulation, stress responses, and tissue repair, positioning MSC-EVs as promising cell-free therapeutic agents. While the overlap of key miRNAs suggests the conservation of stem cell function, the selective enrichment of others highlights the unique capabilities of EVs to modulate recipient cell behaviour. Our results align closely with the existing literature, while refining existing models of therapeutic activity with enhanced reproducibility and in a clinically relevant, methodologically robust context. Future studies should build on these findings by exploring the mechanisms of miRNA sorting, validating predicted targets in functional assays, and refining EV-based therapeutics for specific clinical applications. Despite recent advances, several critical challenges remain. Greater clarity is needed around the definition and standardization of MSC-EV preparations, including how to distinguish MSC-EVs from other vesicle populations, establish meaningful purity thresholds, and define functional integrity. Moreover, it remains uncertain to what extent in vitro-derived MSC-EVs reflect their in vivo counterparts, a question that must be addressed to fully realise their clinical potential [50]. Continued refinement in terms of the isolation, characterization, and functional mechanistic validation will be essential to harness the full therapeutic promise of MSC-EVs.

## 4. Materials and Methods

### 4.1. MSC Culture and Characterization

Human bone-marrow-derived MSCs (BM-MSC) were generated using a standard plastic adherence method from independent healthy donor bone-marrow aspirates that were surplus to hematopoietic stem cell transplantation, as previously described [74]. Samples were collected with informed consent and Local Research Ethical Committee approval (NRES Committee North East—Newcastle and North Tyneside 2, 14/NE/1136) and obtained from the Newcastle Cellular Therapy Facility, Newcastle upon Tyne, UK. MSCs were cultured in a basal medium containing Dulbecco’s modified eagle medium (DMEM), 100 IU/mL penicillin, 100 μg/mL streptomycin, 2 IU/mL heparin, and 2 mM L-glutamine (Sigma-Aldrich, St. Louis, MO, USA), supplemented with 5% human platelet lysate (hPL; PLTMax, Mill Creek Lifesciences, Rochester, MN, USA) (5% hPL/DMEM) and were characterised at passage 3 according to the criteria described by the International Society of Cellular Therapy (ISCT) [8].

### 4.2. MSC-EV Isolation

MSC-EVs were collected from MSC-conditioned medium at passage 3 by differential ultracentrifugation (UC), as previously described [75]. EV-depleted medium was prepared by 18 h UC at 100,000× *g* of 10% hPL/DMEM. At 90% confluence, passage 3 MSCs were washed twice with phosphate buffered saline (PBS, Sigma-Aldrich) and cultured in EV-depleted medium (final concentration: EV-depleted 5% hPL/DMEM), for 48 h prior to MSC-EV isolation. The conditioned medium was subjected to a centrifugation and UC protocol as previously described [74], and the resultant MSC-EV pellet was re-suspended in >100 μL of sterile particle-free PBS and stored at −80 °C.

### 4.3. MSC-EV Characterization

#### 4.3.1. Transmission Electron Microscopy

Transmission electron microscopy [76] was performed using 300-mesh grids (Gilder Grids, Grantham, UK) filmed with Pioloform^®^ (SPI Supplies, West Chester, PA, USA), that were carbon coated and plasma etched before use. Each EV pellet was re-suspended in 100 μL PBS and a 10 µL droplet picked up by each grid, incubated for 5 s, stained with uranyl acetate (Agar Scientific, Rotherham, UK), and air-dried. Grids were examined using a Hitachi HT7800 TEM (Tokyo, Japan) and digital images were collected using an Emsis Xarosa camera with Radius software v2, in conjunction with the Electron Microscopy Research Services (Newcastle University, Newcastle, UK).

#### 4.3.2. Nanoparticle Tracking Analysis

Nanoparticle tracking analysis (NTA) was performed using a NanoSight LM10-HS microscope (Malvern Panalytical Ltd., Worcestershire, UK) equipped with NTA software v2.3 (NanoSight Ltd., Worcestershire, UK). Background extraction was applied and the automatic setting for the minimum expected particle size, minimum track length, and blur settings were employed. Three 60 s recordings were recorded for each sample, diluted at 1:10,000 in sterile filtered PBS (Sigma). Only measurements with >1000 completed tracks were analysed.

#### 4.3.3. Western Blot

Western Blot analyses for Alix and Flotillin-1 were performed by the lysis of EVs (2% sodium dodecyl sulfate (SDS) and manual shearing (1 mL syringe and 0.8 × 38 mm of 21G needle (Terumo, Tokyo, Japan)). Protein quantification was determined using the Micro BCA™ Protein Assay Kit (ThermoFisher, Waltham, MA, USA) following the manufacturer’s directions. Absorbance values were measured using a Microplate Reader (Thermo Labsystems Multiskan Ascent 354) with a 575 nm wavelength. Protein lysates were diluted in Laemmli buffer containing 0.2% bromophenol (Fisher Scientific) and 50 μL/mL of β-merceptoethanol (Sigma) and heated at 95 °C prior to loading onto a 4–20% Mini-PROTEAN^®^ TGX™ Precast Gel (Bio-Rad Laboratories, Hercules, CA, USA) alongside controls and molecular Precision Plus Protein™ Dual Colour Standards (Bio-Rad Laboratories). Blots were incubated with a 1:1000 primary antibody followed by a 1:1500 secondary antibodies (Polyclonal Goat anti-Mouse, Daco). Blots were visualised under chemiluminescence detection using a clarity reagent (Bio-Rad), the LI-COR Odyssey FC Imaging System and Image Studio software v5.2 (LI-COR).

#### 4.3.4. Flow Cytometry

For flow cytometric (FC) assessment of CD64, CD9 and CD81, EVs were coated onto 4 µm aldehyde/sulphate latex beads (ThermoFisher), blocked with 1 M Glycine (Sigma), washed, and then incubated for 2 h at 4 °C with anti-human PE CD63 (H5C6), PerCPCy5.5 CD9 (M-L13), and APC CD81 (JS-81) antibodies or corresponding isotype controls (all from BD Biosciences, San Jose, CA, USA). Data acquisition was performed using a FACS Canto II cytometer (BD Biosciences) and analysed with FlowJo v10.0 software (Tree Star Inc., Ashland, OR, USA).

### 4.4. RNA Isolation and Quantification

Total RNA was isolated from EVs using the Total Exosome and Protein Isolation Kit (Invitrogen, Waltham, MA, USA) and from cells using the miRNeasy Mini Kit (Qiagen, Germantown MD, USA), as per the manufacturer’s instructions. For NanoString profiling, RNA was concentrated to 25 μL, incorporating Amicon Ultra-0.5 Centrifugal Filter Units (Merck Millipore, Burlington, MA, USA). All RNA was quantified using the Bioanalyzer and RNA 6000 Pico kit (Agilent, Santa Clara, CA, USA), RNA 6000 Nano kit (Agilent), or the NanoDrop 1000 spectrophotometer (ThermoFisher), as appropriate.

### 4.5. Quantitative Real-Time PCR

MicroRNA and endogenous control-specific cDNA (HY3 and U6) were generated using TaqMan^®^ MicroRNA Assays or TaqMan^®^ Control Assays and the TaqMan^®^ MicroRNA Reverse Transcription kit (ThermoFisher), according to the supplier’s protocol. Each reaction incorporated TaqMan^®^ MicroRNA Assays or TaqMan^®^ Control Assays (ThermoFisher) and the SensiFast Probe Hi-Rox reagent (Bioline, Alvinston, ON, USA). Thermal cycling was performed in triplicate using the 7900HT Real-Time PCR System (ThermoFisher), according to the manufacturer’s recommended cycling conditions.

### 4.6. NanoString

Total RNA was profiled using the nCounter^®^ Human v4.0 miRNA Expression Assay Kit (NanoString Technologies, Seattle, WA, USA) as previously described [77], based on miRBase v21. The code set incorporated 799 mature microRNAs and included 6 positive controls, 8 negative controls, 6 ligation controls, and 5 mRNA housekeeping controls (*ACTB*, *B2M*, *GAPDH*, *RPL19*, and *RPLP0*). The starting material comprised 5 μL of concentrated serum RNA. Data normalization was performed using nSolver v4.0. Normalised counts were filtered based on >20 counts in >2 samples.

### 4.7. Pathway and Gene Enrichment Analysis

Target KEGG pathways for selected microRNAs were predicted using miRPath v.3 (Diana Tools), based on microT-CDS predicted targets and TarBase v.8 experimentally supported targets, incorporating gene union with *p* < 0.05, microT threshold = 0.8, and FDR correction [76]. Gene canonical pathways, upstream regulators, diseases and functions, and mechanistic networks were performed through the use of Ingenuity Pathway Analysis [71] (Qiagen Inc.). MicroRNA–mRNA pairings were analysed using the IPA MicroRNA Target Filter, based on experimentally validated interactions from TarBase and miRecords, and predicted interactions from TargetScan. Predicted microRNA targets were identified using miRWalk [78], based on TarPmiR, TargetScan, miRDB, and miRTarBase.

## Data Availability

The raw data supporting the conclusions of this article will be made available by the authors on request.

## Supplementary Materials

The following supporting information can be downloaded at: https://www.mdpi.com/article/10.3390/ijms26147010/s1. References [79,80] are cited in the Supplementary Materials.

## References

[1] Uccelli A., Moretta L., Pistoia V. (2008). Mesenchymal stem cells in health and disease. Nat. Rev. Immunol..

[2] Zuk P.A., Zhu M., Mizuno H., Huang J., Futrell J.W., Katz A.J., Benhaim P., Lorenz H.P., Hedrick M.H. (2001). Multilineage cells from human adipose tissue: Implications for cell-based therapies. Tissue Eng..

[3] Griffiths M.J., Bonnet D., Janes S.M. (2005). Stem cells of the alveolar epithelium. Lancet.

[4] Beltrami A.P., Barlucchi L., Torella D., Baker M., Limana F., Chimenti S., Kasahara H., Rota M., Musso E., Urbanek K. (2003). Adult cardiac stem cells are multipotent and support myocardial regeneration. Cell.

[5] Tsai M.S., Lee J.L., Chang Y.J., Hwang S. (2004). Isolation of human multipotent mesenchymal stem cells from second-trimester amniotic fluid using a novel two-stage culture protocol. Hum. Reprod..

[6] Igura K., Zhang X., Takahashi K., Mitsuru A., Yamaguchi S., Takahashi T. (2004). Isolation and characterization of mesenchymal progenitor cells from chorionic villi of human placenta. Cytotherapy.

[7] Wang H.S., Hung S.C., Peng S.T., Huang C.C., Wei H.M., Guo Y.J., Fu Y.S., Lai M.C., Chen C.C. (2004). Mesenchymal stem cells in the Wharton’s jelly of the human umbilical cord. Stem Cells.

[8] Dominici M., Le Blanc K., Mueller I., Slaper-Cortenbach I., Marini F., Krause D., Deans R., Keating A., Prockop D., Horwitz E. (2006). Minimal criteria for defining multipotent mesenchymal stromal cells. The International Society for Cellular Therapy position statement. Cytotherapy.

[9] Castro-Manrreza M.E., Montesinos J.J. (2015). Immunoregulation by mesenchymal stem cells: Biological aspects and clinical applications. J. Immunol. Res..

[10] Martínez-Carrasco R., Sanchez-Abarca L., Nieto-Gomez C., García E.M., Sánchez-Guijo F., Argüeso P., Aijón J., Hernández-Galilea E., Velasco A. (2019). Subconjunctival injection of mesenchymal stromal cells protects the cornea in an experimental model of GVHD. Ocul. Surf..

[11] Bartunek J., Behfar A., Dolatabadi D., Vanderheyden M., Ostojic M., Dens J., El Nakadi B., Banovic M., Beleslin B., Vrolix M. (2013). Cardiopoietic stem cell therapy in heart failure: The C-CURE (Cardiopoietic stem Cell therapy in heart failURE) multicenter randomized trial with lineage-specified biologics. J. Am. Coll. Cardiol..

[12] Shi M., Zhang Z., Xu R., Lin H., Fu J., Zou Z., Zhang A., Shi J., Chen L., Lv S. (2012). Human mesenchymal stem cell transfusion is safe and improves liver function in acute-on-chronic liver failure patients. Stem Cells Transl. Med..

[13] Yun C.W., Lee S.H. (2019). Potential and Therapeutic Efficacy of Cell-based Therapy Using Mesenchymal Stem Cells for Acute/chronic Kidney Disease. Int. J. Mol. Sci..

[14] Ankrum J.A., Ong J.F., Karp J.M. (2014). Mesenchymal stem cells: Immune evasive, not immune privileged. Nat. Biotechnol..

[15] Park Y.B., Ha C.W., Lee C.H., Yoon Y.C., Park Y. (2017). Cartilage Regeneration in Osteoarthritic Patients by a Composite of Allogeneic Umbilical Cord Blood-Derived Mesenchymal Stem Cells and Hyaluronate Hydrogel: Results from a Clinical Trial for Safety and Proof-of-Concept with 7 Years of Extended Follow-Up. Stem Cells Transl. Med..

[16] Volarevic V., Arsenijevic N., Lukic M.L., Stojkovic M. (2011). Concise review: Mesenchymal stem cell treatment of the complications of diabetes mellitus. Stem Cells.

[17] Kim S.J., Moon G.J., Chang W.H., Kim Y.-H., Bang O.Y. (2013). Intravenous transplantation of mesenchymal stem cells preconditioned with early phase stroke serum: Current evidence and study protocol for a randomized trial. Trials.

[18] Okamoto R., Yajima T., Yamazaki M., Kanai T., Mukai M., Okamoto S., Ikeda Y., Hibi T., Inazawa J., Watanabe M. (2002). Damaged epithelia regenerated by bone marrow-derived cells in the human gastrointestinal tract. Nat. Med..

[19] Shan Y., Zhang M., Tao E., Wang J., Wei N., Lu Y., Liu Q., Hao K., Zhou F., Wang G. (2024). Pharmacokinetic characteristics of mesenchymal stem cells in translational challenges. Signal Transduct. Target. Ther..

[20] Sharma D., Zhao F. (2021). Updates on clinical trials evaluating the regenerative potential of allogenic mesenchymal stem cells in COVID-19. npj Regen. Med..

[21] Weiss D.J., Rolandsson Enes S. (2024). MSC-Based Cell Therapy for COVID-19-Associated ARDS and Classical ARDS: Comparative Perspectives. Curr. Stem Cell Rep..

[22] Casiraghi F., Noris M., Remuzzi G. (2010). Immunomodulatory effects of mesenchymal stromal cells in solid organ transplantation. Curr. Opin. Organ Transplant..

[23] Jones S., Horwood N., Cope A., Dazzi F. (2007). The antiproliferative effect of mesenchymal stem cells is a fundamental property shared by all stromal cells. J. Immunol..

[24] Corcione A., Benvenuto F., Ferretti E., Giunti D., Cappiello V., Cazzanti F., Risso M., Gualandi F., Mancardi G.L., Pistoia V. (2006). Human mesenchymal stem cells modulate B-cell functions. Blood.

[25] Glennie S., Soeiro I., Dyson J., Lam E., Dazzi F. (2005). Bone marrow mesenchymal stem cells induce division arrest anergy of activated T cells. Blood.

[26] Nauta A.J., Kruisselbrink A.B., Lurvink E., Willemze R., Fibbe W.E. (2006). Mesenchymal stem cells inhibit generation and function of both CD34+-derived and monocyte-derived dendritic cells. J. Immunol..

[27] Bruno S., Grange C., Deregibus M.C., Calogero R.A., Saviozzi S., Collino F., Morando L., Busca A., Falda M., Bussolati B. (2009). Mesenchymal stem cell-derived microvesicles protect against acute tubular injury. J. Am. Soc. Nephrol..

[28] Kim S.J., Moon G.J., Cho Y.H., Kang H.Y., Hyung N.K., Kim D., Lee J.H., Nam J.Y., Bang O.Y., Rameshwar P. (2012). Circulating mesenchymal stem cells microparticles in patients with cerebrovascular disease. PLoS ONE.

[29] Lai R.C., Arslan F., Tan S.S., Tan B., Choo A., Lee M.M., Chen T.S., Teh B.J., Eng J.K.L., Sidik H. (2010). Derivation and characterization of human fetal MSCs: An alternative cell source for large-scale production of cardioprotective microparticles. J. Mol. Cell. Cardiol..

[30] Keerthikumar S., Chisanga D., Ariyaratne D., Al Saffar H., Anand S., Zhao K., Samuel M., Pathan M., Jois M., Chilamkurti N. (2016). ExoCarta: A Web-Based Compendium of Exosomal Cargo. J. Mol. Biol..

[31] Lai R.C., Yeo R.W., Lim S.K. (2015). Mesenchymal stem cell exosomes. Semin. Cell Dev. Biol..

[32] Zhang B., Yin Y., Lai R.C., Tan S.S., Choo A.B.H., Lim S.K. (2014). Mesenchymal stem cells secrete immunologically active exosomes. Stem Cells Dev..

[33] Stawarska A., Bamburowicz-Klimkowska M., Runden-Pran E., Dusinska M., Cimpan M.R., Rios-Mondragon I., Grudzinski I.P. (2024). Extracellular Vesicles as Next-Generation Diagnostics and Advanced Therapy Medicinal Products. Int. J. Mol. Sci..

[34] Milbank E., Cragano N.R.V., Gonzalez-Garcia I., Garcia M.R., Rivas-Limeres V., Perdomo L., Hilairet G., Ruiz-Pino F., Mallegol P., Morgan D.A. (2021). Small extracellular vesicle-mediated targeting of hypothalamic AMPKα1 corrects obesity through BAT activation. Nat. Metab..

[35] Watanabe Y., Tsuchiya A., Terai S. (2021). The development of mesenchymal stem cell therapy in the present, and the perspective of cell-free therapy in the future. Clin. Mol. Hepatol..

[36] Herrmann I.K., Wood M.J.A., Fuhrmann G. (2021). Extracellular vesicles as a next-generation drug delivery platform. Nat. Nanotechnol..

[37] Phinney D.G., Di Giuseppe M., Njah J., Sala E., Shiva S., St Croix C.M., Stolz D.B., Watkins S.C., Di Y.P., Leikauf G.D. (2015). Mesenchymal stem cells use extracellular vesicles to outsource mitophagy and shuttle microRNAs. Nat. Commun..

[38] Baglio S.R., Rooijers K., Koppers-Lalic D., Verweij F.J., Pérez Lanzón M., Zini N., Naaijkens B., Perut F., Niessen H.W.M., Baldini N. (2015). Human bone marrow- and adipose-mesenchymal stem cells secrete exosomes enriched in distinctive miRNA and tRNA species. Stem Cell Res. Ther..

[39] Jothimani G., Pathak S., Dutta S., Duttaroy A.K., Banerjee A. (2022). A Comprehensive Cancer-Associated MicroRNA Expression Profiling and Proteomic Analysis of Human Umbilical Cord Mesenchymal Stem Cell-Derived Exosomes. Tissue Eng. Regen. Med..

[40] Soni N., Gupta S., Rawat S., Krishnakumar V., Mohanty S., Banerjee A. (2022). MicroRNA-Enriched Exosomes from Different Sources of Mesenchymal Stem Cells Can Differentially Modulate Functions of Immune Cells and Neurogenesis. Biomedicines.

[41] Nakamura Y., Miyaki S., Ishitobi H., Matsuyama S., Nakasa T., Kamei N., Akimoto T., Higashi Y., Ochi M. (2015). Mesenchymal-stem-cell-derived exosomes accelerate skeletal muscle regeneration. FEBS Lett..

[42] Liu Y., Zou R., Wang Z., Wen C., Zhang F., Lin F. (2018). Exosomal KLF3-AS1 from hMSCs promoted cartilage repair and chondrocyte proliferation in osteoarthritis. Biochem. J..

[43] Shao L., Zhang Y., Lan B., Wang J., Zhang Z., Zhang L., Xiao P., Meng Q., Geng Y.-j., Yu X.-y. (2017). MiRNA-Sequence Indicates That Mesenchymal Stem Cells and Exosomes Have Similar Mechanism to Enhance Cardiac Repair. BioMed Res. Int..

[44] Hemeda H., Giebel B., Wagner W. (2014). Evaluation of human platelet lysate versus fetal bovine serum for culture of mesenchymal stromal cells. Cytotherapy.

[45] Haque N., Kasim N.H., Rahman M.T. (2015). Optimization of pre-transplantation conditions to enhance the efficacy of mesenchymal stem cells. Int. J. Biol. Sci..

[46] Spees J.L., Gregory C.A., Singh H., Tucker H.A., Peister A., Lynch P.J., Hsu S.-C., Smith J., Prockop D.J. (2004). Internalized antigens must be removed to prepare hypoimmunogenic mesenchymal stem cells for cell and gene therapy. Mol. Ther..

[47] Crespo-Diaz R., Behfar A., Butler G.W., Padley D.J., Sarr M.G., Bartunek J., Dietz A.B., Terzic A. (2011). Platelet lysate consisting of a natural repair proteome supports human mesenchymal stem cell proliferation and chromosomal stability. Cell Transplant..

[48] Bernardo M.E., Avanzini M.A., Perotti C., Cometa A., Moretta A., Lenta E., Del Fante C., Novara F., de Silvestri A., Amendola G. (2007). Optimization of in vitro expansion of human multipotent mesenchymal stromal cells for cell-therapy approaches: Further insights in the search for a fetal calf serum substitute. J. Cell. Physiol..

[49] Flemming A., Schallmoser K., Strunk D., Stolk M., Volk H.-D., Seifert M. (2011). Immunomodulative efficacy of bone marrow-derived mesenchymal stem cells cultured in human platelet lysate. J. Clin. Immunol..

[50] Witwer K.W., Van Balkom B.W.M., Bruno S., Choo A., Dominici M., Gimona M., Hill A.F., De Kleijn D., Koh M., Lai R.C. (2019). Defining mesenchymal stromal cell (MSC)-derived small extracellular vesicles for therapeutic applications. J. Extracell. Vesicles.

[51] Welsh J.A., Goberdhan D.C.I., O’Driscoll L., Buzas E.I., Blenkiron C., Bussolati B., Cai H., Di Vizio D., Driedonks T.A.P., Erdbrügger U. (2024). Minimal information for studies of extracellular vesicles (MISEV2023): From basic to advanced approaches. J. Extracell. Vesicles.

[52] Vaka R., Parent S., Risha Y., Khan S., Courtman D., Stewart D.J., Davis D.R. (2023). Extracellular vesicle microRNA and protein cargo profiling in three clinical-grade stem cell products reveals key functional pathways. Mol. Ther.—Nucleic Acids.

[53] Zou X.Y., Yu Y., Lin S., Zhong L., Sun J., Zhang G., Zhu Y. (2018). Comprehensive miRNA Analysis of Human Umbilical Cord-Derived Mesenchymal Stromal Cells and Extracellular Vesicles. Kidney Blood Press. Res..

[54] Zhou Y., Ochiya T., Xiao Z., Itaya T. (2018). Distinct Mirna Expression Patterns of Extracellular Vesicles Derived From 4 Types of Mesenchymal Stem Cells. J. Stem Cell Res. Ther..

[55] Chen T.S., Lai R.C., Lee M.M., Choo A.B.H., Lee C.N., Lim S.K. (2009). Mesenchymal stem cell secretes microparticles enriched in pre-microRNAs. Nucleic Acids Res..

[56] Ferguson S.W., Wang J., Lee C.J., Liu M., Neelamegham S., Canty J.M., Nguyen J. (2018). The microRNA regulatory landscape of MSC-derived exosomes: A systems view. Sci. Rep..

[57] Furuta T., Miyaki S., Ishitobi H., Ogura T., Kato Y., Kamei N., Miyado K., Higashi Y., Ochi M. (2016). Mesenchymal Stem Cell-Derived Exosomes Promote Fracture Healing in a Mouse Model. Stem Cells Transl. Med..

[58] Figueroa J., Phillips L.M., Shahar T., Hossain A., Gumin J., Kim H., Bean A.J., Calin G.A., Fueyo J., Walters E.T. (2017). Exosomes from Glioma-Associated Mesenchymal Stem Cells Increase the Tumorigenicity of Glioma Stem-like Cells via Transfer of miR-1587. Cancer Res..

[59] Zubkova E., Evtushenko E., Beloglazova I., Osmak G., Koshkin P., Moschenko A., Menshikov M., Parfyonova Y. (2021). Analysis of MicroRNA Profile Alterations in Extracellular Vesicles From Mesenchymal Stromal Cells Overexpressing Stem Cell Factor. Front. Cell Dev. Biol..

[60] Dos Santos C.C., Lopes-Pacheco M., English K., Rolandsson Enes S., Krasnodembskaya A., Rocco P.R.M. (2024). The MSC-EV-microRNAome: A Perspective on Therapeutic Mechanisms of Action in Sepsis and ARDS. Cells.

[61] Nazari-Shafti T.Z., Neuber S., Duran A.G., Exarchos V., Beez C.M., Meyborg H., Krüger K., Wolint P., Buschmann J., Böni R. (2020). MiRNA Profiles of Extracellular Vesicles Secreted by Mesenchymal Stromal Cells-Can They Predict Potential Off-Target Effects?. Biomolecules.

[62] Sanjurjo-Rodríguez C., Crossland R.E., Reis M., Pandit H., Wang X.-n., Jones E. (2021). Characterization and miRNA Profiling of Extracellular Vesicles from Human Osteoarthritic Subchondral Bone Multipotential Stromal Cells (MSCs). Stem Cells Int..

[63] Hu S., Zhang C., Ma Q., Li M., Yu X., Zhang H., Lv S., Shi Y., He X. (2024). Unveiling the multifaceted roles of microRNAs in extracellular vesicles derived from mesenchymal stem cells: Implications in tumor progression and therapeutic interventions. Front. Pharmacol..

[64] Wang X., Mijiti W., Jia Q., Yi Z., Ma J., Zhou Z., Xie Z. (2024). Exploration of altered miRNA expression and function in MSC-derived extracellular vesicles in response to hydatid antigen stimulation. Front. Microbiol..

[65] Chang C.C., Venø M.T., Chen L., Ditzel N., Le D.Q.S., Dillschneider P., Kassem M., Kjems J. (2018). Global MicroRNA Profiling in Human Bone Marrow Skeletal-Stromal or Mesenchymal-Stem Cells Identified Candidates for Bone Regeneration. Mol. Ther..

[66] Kou M., Huang L., Yang J., Chiang Z., Chen S., Liu J., Guo L., Zhang X., Zhou X., Xu X. (2022). Mesenchymal stem cell-derived extracellular vesicles for immunomodulation and regeneration: A next generation therapeutic tool?. Cell Death Dis..

[67] Kageyama Y., Koide Y., Yoshida A., Uchijima M., Arai T., Miyamoto S., Ozeki T., Hiyoshi M., Kushida K., Inoue T. (1998). Reduced susceptibility to collagen-induced arthritis in mice deficient in IFN-gamma receptor. J. Immunol..

[68] Huang C.C., Kang M., Leung K., Lu Y., Shirazi S., Gajendrareddy P., Ravindran S. (2023). Micro RNA based MSC EV engineering: Targeting the BMP2 cascade for bone repair. Front. Cell Dev. Biol..

[69] Park J.H., Choi Y., Lim C.W., Park J.M., Yu S.H., Kim Y., Han H.J., Kim C.H., Song Y.S., Kim C. (2021). Potential Therapeutic Effect of Micrornas in Extracellular Vesicles from Mesenchymal Stem Cells against SARS-CoV-2. Cells.

[70] Crossland R.E., Albiero A., Sanjurjo-Rodríguez C., Reis M., Resteu A., Anderson A.E., Dickinson A.M., Pratt A.G., Birch M., McCaskie A.W. (2023). MicroRNA profiling of low concentration extracellular vesicle RNA utilizing NanoString nCounter technology. J. Extracell. Biol..

[71] Srivastava J., Kundal K., Rai B., Saxena P., Katiyar S., Tripathy N., Yadav S., Gupta R., Kumar R., Nityanand S. (2024). Global microRNA profiling of bone marrow-MSC derived extracellular vesicles identifies miRNAs associated with hematopoietic dysfunction in aplastic anemia. Sci. Rep..

[72] Wang X., Omar O., Vazirisani F., Thomsen P., Ekström K. (2018). Mesenchymal stem cell-derived exosomes have altered microRNA profiles and induce osteogenic differentiation depending on the stage of differentiation. PLoS ONE.

[73] Eirin A., Zhu X.-Y., Puranik A.S., Woollard J.R., Tang H., Dasari S., Lerman A., van Wijnen A.J., Lerman L.O. (2017). Integrated transcriptomic and proteomic analysis of the molecular cargo of extracellular vesicles derived from porcine adipose tissue-derived mesenchymal stem cells. PLoS ONE.

[74] Reis M., Mavin E., Nicholson L., Green K., Dickinson A.M., Wang X.N. (2018). Mesenchymal Stromal Cell-Derived Extracellular Vesicles Attenuate Dendritic Cell Maturation and Function. Front. Immunol..

[75] Théry C., Amigorena S., Raposo G., Clayton A. (2006). Isolation and characterization of exosomes from cell culture supernatants and biological fluids. Curr. Protoc. Cell Biol..

[76] Vlachos I.S., Zagganas K., Paraskevopoulou M.D., Georgakilas G., Karagkouni D., Vergoulis T., Dalamagas T., Hatzigeorgiou A.G. (2015). DIANA-miRPath v3.0: Deciphering microRNA function with experimental support. Nucleic Acids Res..

[77] Crossland R.E., Norden J., Juric M.K., Green K., Pearce K.F., Lendrem C., Greinix H.T., Dickinson A.M. (2017). Expression of Serum microRNAs is Altered During Acute Graft-versus-Host Disease. Front. Immunol..

[78] Sticht C., De La Torre C., Parveen A., Gretz N. (2018). miRWalk: An online resource for prediction of microRNA binding sites. PLoS ONE.

[79] Fang S., Xu C., Zhang Y., Xue C., Yang C., Bi H., Qian X., Wu M., Ji K., Zhao Y. (2016). Umbilical Cord-Derived Mesenchymal Stem Cell-Derived Exosomal MicroRNAs Suppress Myofibroblast Differentiation by Inhibiting the Transforming Growth Factor-β/SMAD2 Pathway During Wound Healing. Stem Cells Transl. Med..

[80] Nakamura A., Rampersaud Y.R., Sharma A., Lewis S.J., Wu B., Datta P., Sundararajan K., Endisha H., Rossomacha E., Rockel J.S. (2016). Identification of microRNA-181a-5p and microRNA-4454 as mediators of facet cartilage degeneration. JCI Insight.

