# Supplementary material for: Selective MicroRNA Packaging Reveals Distinct Core Signatures in Human Mesenchymal-Stromal-Cell-Derived Extracellular Vesicles
Source: Int J Mol Sci. 2025 Jul 21;26(14):7010. doi: 10.3390/ijms26147010 (PMC12295167; doi:10.3390/ijms26147010)
Supplement: Supplementary file 1 [file ijms-26-07010-s001.zip › ijms-3720403-supplementary.pdf]

Author, Year, Reference	Source of MSC	No. of MSC donors	MSC culture media	No. of Cells for EVs	MSC media for EV Isolation	EV Isolation method	EV characterisation	MicroRNA Profiling method	MSC/MSC-EV MicroRNAs
**Phinney *et al*, 2015 [37]**	Human BM, mouse	5 human donors	Medium depleted of serum derived-MVs/EVs or in serum-reduced MSC medium with BSA	Batches of 10 × 106 MSCs	Serum-reduced MSC medium with BSA	UC and sucrose cushion	CD9, CD63 and TSG101 expression, NTA	miRNeasy Mini Kit, Agilent human microRNA microarrays	156 (45 increased; 111 decreased) microRNAs differed in abundance between EVs vs. MSCs. 10 microRNAs with the greatest increase included miR-451a, -1202, -630 and -638, while microRNAs with the greatest decrease in EVs and enriched in MSCs included miR-125b and miR-21. Pattern was conserved in MSC-EVs from 5 human donors.
**Baglio *et al*, 2015 [38]**	Human AT	AT n=3, BM n=4	alpha-MEM with 100 U/ml penicillin, 100 μg/ml streptomycin, and 10 % FBS or 5 % PL and 10 U/ml heparin	3.2 × 107 cells at early passages (passages 2–3)	α-MEM containing exosome-depleted FBS or PL for 24-48 h	UC	TEM, CD63 & CD81 by WB	Trizol, RNase A, TruSeq Small RNA Sample Preparation Kit, HiSeq 2000 paired end 100 cycle.	Five most abundant miRNAs (miR-486-5p, -10a-5p, -10b-5p, -191-5p, -222-3p in ASC EVs; miR-143-3p, -10b-5p, -486-5p, -22-3p, -21-5p in BMSC EVs) accounted for 43–59 % of total miRNA reads. MiR-21-5p, -22-3p, -10b-5p, -222-3p were among the most represented in both cells and EVs. MiR-4485, -150-5p, -6087 and -486-5p overrepresented in EVs vs. MSCs. MiR-34a-5p, -34c-5p, -15a-5p, -136-3p significantly overrepresented in cells vs. EVs.
**Jothimani *et al*, 2022 [39]**	Human UC		Low glucose DMEM with 20% FBS, L-glutamax, and 1% antibiotic–antimycotic solution		DMEM supplemented with exosome depleted FBS for 72 h	Total Exosome Isolation reagent	CD63 expression, EM, DLS (Nano ZS90)	miRNeasy Micro Kit, miScript RT II kit, MIHS 102ZD-Human Cancer Pathway Finder and miScript SYBR green PCR kit.	High expression of miR-21-5p, -125b-5p, -146a-5p, -29a-3p, -27a-3p, -100-5p, -143-3p, -222-3p and let-7 family miRNAs in hUCMSCs and hUCMSC-EVs. Both hUCMSCs and hUCMSC-EVs lacked or had less expression of miRNAs miR-96-5p, -184, -183-5p, -373-3p, -144- 3p, -372-3p, -206.

**Soni *et al,* 2022 [40]**	Human BM, AT & WJ	n=4 for each MSC type	BM-MSC DMEM-LG + 10% FBS and 1% P/S; AD-MSC DMEM-LG + P/S + Gentamycin; WJ-MSC α-MEM + 15% human serum + L-glutamine + kanamycin		Serum-free CM for 48 h	Sucrose cushion UC	NTA, TEM, WB for CD63 and Alix, BCA	miRCURY RNA Isolation kit, Illumina Hi Seq 2500	193 (61.3%) microRNAs expressed in all MSC. 122 (38.7%) expressed in all MSC and all EV. Expression pattern of miRNAs in EVs was different to parental hMSCs. Except for miR-143-5p and 181c-5p in BM, and miR-146a-5p in WJ, the expression of most miRNAs in EVs was reduced vs. MSC. Expression of miRNAs in EVs was different to parental hMSCs. Except for miR-143-5p and 181c-5p in BM, and miR-146a-5p in WJ, the expression of most miRNAs in EVs was reduced vs. MSC.
**Shao *et al,* 2017 [43]**	Bone derived from male Sprague-Dawley rats		DMEM : nutrient mixture F-12 (DMEM : F12, HyClone) with 10% FBS, 100 U/mL penicillin, 100 µg/mL streptomycin		MSCs in DMEM:F12 with 10% FBS. The FBS centrifuged at 100,000 to eliminate preexisting bovine-EVs.	Total exosome isolation kit	BCA, TEM, CD63 by FC and WB	HiSeq 2500 platform	MSC-Exo and MSCs had similar miRNA sequence profile in general. 20 microRNAs were significantly DE, including miR-15a-3p.
**Vaka *et al,* 2023 [52]**	Human BM and CB		BM-MSCs NutriStem XF media under 21% oxygen condition. UC-MSCs DMEM with 10% PL		(BM-MSCs and HDCs: NutriStem XF basal media; UC-MSCs: DMEM with high glucose and 1% platelet lysate for 48 h	UC	NTA (NanoSight LM10), proteomic array (Exoray200a)	microRNA isolation kit, Nanostring nCounter microRNA assay.	757 microRNAs commonly expressed by all 3 EVs. Highest expression of miR-199a+miR-199b, miR-23a, miR-4454+miR-7975, miR-125b-5p in all EVs.
**Zhou *et al,* 2018 [54]**	Human BM, AT, WJ & SHED	4 types of MSC, donated by n=4 donors.	DMEM D6429 + Penicillin + streptomycin + amphotericin B + 10% FBS		Serum-free DMEM D6429 for 48 h prior to EV isolation.	UC	NTA, BA	QIAzol reagent and the miRNeasy Mini Kit. Agilent SurePrint G3 Human miRNA 8x60K array	91 miRNAs commonly detected in 4 types of MSC-EVs, including miR-199a-3p, -24-3p, -29a-3p, -23a-3p, -638, -125b-5p, -630, -21-5p. 4, 25, 29 and 35 miRNAs were selected as cell-type specific miRNAs in EVs derived from AT-MSC, WJ-MSC, SHED and BM- MSC, respectively.

**Zou *et al*, 2018 [53]**	Human UC		DMEM low glucose with 10% FBS		DMEM low glucose for overnight incubation	UC	CD81 and CD9 expression	Qiagen RNA isolation kit, miRCURYTM Array Power Labeling Kit, Axon GennePix 4000B Microarray Scanner	MSCs and EVs expressed similar amounts of miRNA (n=2089). Only 0.8% (16/2089) and 0.9% (19/2089) of miRNAs were enriched in MSCs and EVs. Most miRNAs (98.3%) showed same expression in both MSCs and EVs, with 0.8% (16/2089) enriched in MSCs and 0.9% (19/2089) enriched in EVs. 16 miRNAs were enriched in MSCs and 20 miRNAs enriched in EVs.
**Chen *et al*, 2009 [55]**	hESC-derived HuES9.E1 MSCs		Serum-free CM		Serum-free CM	Ultrafiltration		Trizol and *mir*Vana™ miRNA Isolation Kit, LC Sciences Microarray	9/13 members of the let-7 family were expressed in MSCs (let-7a/-7b/-7c/-7d/-7e/-7f/-7g/-7i/miR-98). Only let-7a/-7b/-7c/-7d were detected in the CM. MSC expressed >151 miRNAs. 60 miRNAs were detected in the CM. 45 were also found in MSCs.
**Ferguson *et al*, 2018 [56]**	Human BM (ATCC® PCS-500-012)				EV-free media 48 hr prior to EV isolation	Total Exosome Isolation Reagent	NTA, TEM, CD63 expression	NanoString	171 microRNAs detected. The top 23 miRNAs accounted for 79.1% of total miRNAs present in MSC EVs.
**Furuta *et al*, 2016 [57]**	Human BM		DMEM with 10% FBS and 1% antibiotic-antimycotic solution	1.0 3 105 per well	Serum-free DMEM for 48 hr	UC and concentrated using Amicon filters	Flotillin-1, CD9 and CD81 expression	NanoString	Of the top 10 microRNAs, miR-4532, miR-125b-5p, and miR-4516 also appeared in the top 30 most highly expressed miRNAs in MSC-EVs. MiR-338-3p and miR-548aa were expressed >3x higher in MSC-EVs vs. CM-Exo.
**Figueroa *et al*, 2017 [58]**	Glioma-associated human MSCs (GA-hMSC).		Eagle Minimum Essential Medium alpha with 10% FBS, 1% penicillin–streptomycin, and 1% glutamine		GA-hMSCs or GSCs were washed and incubated for 48 hours in serum-free and supplement-free NSC medium.	dUC	CD63 by WB, TEM	mirVana RNA Isolation Kit, miRNA microarray (µParaflo microfluidic biochip technology	According to top 20 differentially expressed miRNAs, EV miRNA cluster separately from cellular miRNA. Enriched microRNA were miR-4530, -1587, -3620-5p, -4507, -4508, -4492, -4505, -1246. Depleted EVs were miR-376c-3p, -9-3p, -103a-3p, -107, -9-5p, -4521, -4284, -199a-5p. 37 EV miRNAs with a ≥5,000 hybridization intensity (top 0.2% of the most highly expressed miRNA) were among EVs derived from all 4 GA-hMSCs.
**Zubkova *et al*, 2021 [59]**	Adipose MSC from Male Wistar-Kyoto rat		D-glucose DMEM with 10% FBS		DMEM/F-12 with 1% EV-depleted FBS for 48 h	dUC	TEM, BA, NTA (NanoSight LM10)	miRCURY RNA Isolation kit, Affymetrix GeneChipTM miRNA Arrays 4.0	1,250 microRNAs detected. 300 miRs downregulated in EV vs. cells. MicroRNA more abundant in MSC vs. EV. Clear division in miRNA expression evident between the MSC vs. EV.

**Nazari-Shafti *et al*, 2020 [61]**	Human AT and CB	AT n=4, CB n=4	MesenPRO RS medium with 10% FBS, 1% P/S, 2ng/mL FGF-b	for UC: 3 × 107 cells at early passages (passages 5–7). For ExoEasy: 3 × 106 cells at early passages	(DMEM 1X)-GlutaMAX, 10% EV depleted FBS, 1% P/S, and 2 ng/mL FGF-b for 48 h, followed by starvation medium (DMEM 1X-GlutaMAX supplemented with 1% P/S and 2 ng/mL FGF-b) for 24 h.	Sequential UC or exoEasy Maxi Kit	Particle concentration and size distribution using ZetaView, protein content using BCA, morphology using TEM, surface CD63/CD9/CD81 by FC	miRNeasy mini kit, miRCURY LNA Universal RT microRNA PCR system with 752 human miRNAs, 3 calibrators and 1 spike-in miRNA internal control	205 microRNAs in all MSC-EV. 66 miRNAs in EVs from both MSC sources. 10 miRNA were uniquely highly expressed in CB-MSC-EVs; let-7d-5p, miR-30a-5p, -106b-5p, -107, -136-5p, -140-3p, -181b-5p, -320b, -320c, -342-3p. 14 were uniquely highly expressed in AT-MSC-EVs; miR-10b-5p, -29b-3p, -138-5p, -148a-3p, -185-5p, -210-3p, -424-3p, -424-5p, -433-3p, -484, -503-5p, -663b, -874-3p, -940. CB-EVs and AT-MSC-EVs are similar in their miRNA composition, with the exception of a small number of miRNAs.
**Wang *et al*, 2018 [72]**	Human BM (ATCC)		EV-free medium prepared according to Thery et al.		EV-free medium	dUC with filtration	TEM, NTA (NanoSight LM10/14), CD63 expression	miRCURY™ RNA Isolation Kit, miRCURY LNA™ Universal RT microRNA PCR Human panel I containing 372 microRNAs	74%, 76% and 70% of sorted microRNAs were shared between EVs and their respective parent cells.
**Eirin *et al*, 2017 [73]**	Porcine primary adipose MSCs		Advanced MEM medium with PL (PLTmax)	10x106 cells		UC	TEM, CD9 and CD63 by WB, NTA	TruSeq	413 miRNAs detected. MiR-183, -378, -140-3p, -222 enriched in EVs vs. MSCs .
**Fang *et al*, 2016 [79]**	Human UC		CMRL with 10% FBS, 2% antibiotic-antimycotic solution, and 1% L-glutamine	Not known	Before isolation, the FBS used was depleted of host exosomes by ultracentrifugation at 120,000*g* for 3 hours at 4°C.	dUC	BCA, NTA, CD81 by WB	High-throughput sequencing	uMSC-Exos had a specific miRNA abundance signature different to HEK293T-Exos and uMSCs. Among the most abundant 10 miRNAs in the uMSC-Exos, only miR-21 was also highly expressed in uMSCs.
**Nakamura *et al*, 2015 [80]**	Human BM	4 independent donors	DMEM with 10% FBS and 1% antibiotic-antimycotic solution	1.0 × 105/well	Serum-free DMEM for 48hr	UC and concentrated using Amicon filters	Flotillin-1, CD9 and CD81 expression, TRPS	NanoString	MiR-21 was detected at the highest concentrations in MSC-EVs. MiRNAs related to tissue regeneration, such as antiapoptotic and antioxidant miRNAs, were present in the MSC-EVs. Other myogenic miRNAs were also detected in EVs, including miR-1, miR-133, and miR-206. Several miRNAs were identified only in EVs and

									miRNAs such as miR-494 was contained abundantly.

Supplementary Table S1. Summary of the literature describing MSC-EV MicroRNA Profiling Studies. AT = Adipose tissue; BM = Bone marrow; BSA = Bovine serum albumin; CMRL = Connaught Medical Research Laboratories developed medium; DMEM = Dulbecco's Modified Eagle Medium; EVs = Extracellular vesicles; FBS = Foetal bovine serum; MEM = minimum essential medium; MSC = Mesenchymal stromal cell; NTA = Nanoparticle tracking analysis; PL = Platel lysate; SHED = stem cells of human exfoliated deciduous teeth; TEM = Transmission electron microscopy; TRPS = Tunable resistance pulse sensing; UC = umbilical cord; UC = ultracentrifugation; WB = Western blot; WJ – Whartons jelly.
